# Serum inflammatory cytokines in the progression of depression

**DOI:** 10.3389/fimmu.2026.1808989

**Published:** 2026-05-13

**Authors:** Qing Feng, Zhen Yuan, Qi An, Kang Wang, Xiaohui Liu

**Affiliations:** 1 Department of Psychology, Air Force 986 Hospital, Xijing Hospital, Fourth Military Medical University, Xi’an, China; 2 Department of Respiratory and Critical Care Medicine, The First Affiliated Hospital of Army Medical University, Chongqing, China

**Keywords:** depression, IL-1β, inflammatory cytokines, neuroimmune interaction, neuroinflammation, TNF-α

## Abstract

Depression is increasingly recognized as a multifactorial disorder in which immune dysregulation contributes substantially to disease initiation, progression, and treatment response. Among the immune mediators implicated, serum inflammatory cytokines—including IL-1β, IL-6, TNF-α, IFN-γ, and C-reactive protein—have emerged as key links between peripheral inflammation and central nervous system dysfunction. These cytokines influence depression-related pathophysiology by activating innate immune signaling pathways, including TLR4, NF-κB, MAPK, and the NLRP3 inflammasome, while also reshaping tryptophan–kynurenine metabolism through IDO1 and TDO2. The resulting alterations impair monoaminergic neurotransmission, enhance glutamatergic excitotoxicity, suppress BDNF-dependent neuroplasticity, and promote microglia-mediated neuroinflammation. Clinical studies further associate inflammatory cytokine profiles with symptom severity, cognitive dysfunction, suicidality, and illness chronicity, supporting their potential value as biologically informative markers in depression. Emerging therapeutic evidence indicates that anti-inflammatory bioactive compounds, conventional antidepressants with immunomodulatory properties, and rapid-acting agents such as ketamine may partially exert their effects by normalizing cytokine-associated pathways. This review summarizes current mechanistic and clinical evidence linking serum inflammatory cytokines to depression and highlights their potential as therapeutic targets in precision psychiatry.

## 1 Introduction

Depression is a chronic neurofunctional disorder primarily characterized by affective disturbances, most commonly including persistent low mood, anhedonia, feelings of hopelessness or worthlessness, emotional blunting, and, in some individuals, irritability or heightened sensitivity to negative stimuli. Importantly, unlike bipolar disorder, depression does not involve episodes of euphoria, mania, or hypomania, which distinguishes its affective profile from other major mood disorders. It often presents insidiously, follows a prolonged clinical course, and is marked by a high risk of relapse, elevated suicide rates, and persistently low rates of diagnosis and treatment engagement (1, 2). Globally, depression contributes to approximately 720, 000 suicide-related deaths each year, representing a significant public health concern and imposing a substantial socio-economic burden (3, 4).

Despite extensive investigation, the etiology and pathophysiology of depression remain incompletely defined. While monoaminergic dysfunction, neuroendocrine dysregulation, and impaired neuroplasticity have been proposed, the immune-inflammatory hypothesis has garnered increasing attention (1, 5). Kronfol et al. (6) reported elevated peripheral leukocyte counts in depression, suggesting immune involvement. Ghaemi (7) subsequently formalized the “immune-inflammatory cytokine hypothesis, ” positing that depression involves a chronic low-grade inflammatory state. Elevated levels of immune-inflammatory cytokines, including IL-1, IL-6, IFNs, TNF-α, and C-reactive protein (CRP), have been observed in multiple studies and are believed to contribute to both the onset and severity of depressive symptoms (8, 9). In the central nervous system, these cytokines activate indoleamine 2, 3-dioxygenase, reducing glutamate reuptake and increasing its release, which in turn suppresses brain-derived neurotrophic factor (BDNF) synthesis. Importantly, Low BDNF levels play a well-established role in depression (10, 11). These cytokines may also modulate the efficacy of antidepressant treatments, serving as potential mediators of therapeutic resistance (**Table 1**).

**Table 1:** Overview of serum inflammatory cytokines in depression.

Category	Cytokines	Mechanisms & signaling pathways	Clinical value	Therapeutic strategy & targeting agents
Neurochemical Imbalance	TNF-α, IFN-γ, IL-1β, IL-6	Activates IDO1/TDO2, shunts Tryptophan to Kynurenine Pathway, reduces Serotonin, modulates glutamate (NMDA R).	Correlates with severity, chronicity, and suicidality; predicts treatment resistance (TNF-α).	Conventional Antidepressants (SSRIs, TCAs): Reduce release; Natural Anti-inflammatory Agents (Rg1, S-equol): Restore Kynurenine balance; Ketamine: Inhibits LHb firing, blocks NMDARs.
Neuroinflammation	IL-1β, IL-18, IL-6	Activates PRRs (TLRs, NLRs), triggers NF-κB, MAPK, and NLRP3 Inflammasome/Caspase-1 signaling.	CRP (>3 mg/L): Linked to low mood, anhedonia, fatigue, sleep/appetite disturbances.	NLRP3/Caspase-1 Inhibitors (Potential); Amitriptyline: Reduces inflammasome expression; Fluoxetine: Inhibits JAK/STAT3 and TLR4/JNK pathways.
Neurotrophic Dysfunction	IL-1β, TNF-α, IL-6	Activated Microglia release cytokines, suppress BDNF synthesis, impair hippocampal neurogenesis/plasticity.	IL-6: Mediator of cognitive deficits (e.g., psychomotor speed, executive flexibility).	GPR55 Agonists (Potential): Counteract IL-1β-induced neurogenesis suppression; HT: Upregulates hippocampal BDNF; Colchicine: Reduces neuronal apoptosis.
Neuroendocrine Dysregulation	IL-1β, IL-6, TNF-α, Cortisol	Elevated Glucocorticoids (CORT) reduce PC12 cell viability, increase ATP stress and DNA damage, upregulate cytokines.	Related to cardiovascular disease risk in aging adults; shows gender-specific vulnerability (higher in females).	NSAIDs (Aspirin): COX-2 inhibition for immune-mediated neuroinflammation (adjunctive); Conventional Antidepressants: Attenuate stress-induced cytokines.
Systemic & Genetic Factors	IL-1β, TNF-α, IL-6	Translates systemic signals via humoral (BBB) and neural (Vagus nerve) pathways; involves gut-brain communication.	IL-1β (Child-Mother correlation): Potential hereditary component in mood disorder susceptibility.	S-equol: Metabolite targeting tryptophan-kynurenine pathway imbalance; Gut-brain axis modulation (Potential): Targets source of systemic inflammation.

Anti-inflammatory agents can ameliorate depressive symptoms by targeting neuroinflammatory processes. Some studies suggest that nonsteroidal anti-inflammatory drugs (aspirin), alone or as adjuncts to conventional antidepressants, enhance therapeutic efficacy, likely by attenuating immune-mediated neuroinflammation via COX-2 inhibition (5, 12). In sum, these findings underscore the potential of inflammatory cytokines as biomarkers for the diagnosis and treatment of depression. Elucidating the role of inflammation in the pathophysiology of depression may provide a theoretical framework for the development of precision medicine approaches. This review summarizes current insights into the involvement of serum inflammatory factors in the onset, diagnosis, and treatment of depression, and to explore how targeting these pathways may improve therapeutic outcomes and reduce depression burden.

## 2 Depression and serum inflammatory cytokines

Elevated serum levels of inflammatory cytokines play a pivotal role in the pathogenesis of depression by disrupting key neurochemical and metabolic pathways (8, 9). These cytokines activate rate-limiting enzymes indoleamine 2, 3-dioxygenase 1 (IDO1) and tryptophan 2, 3-dioxygenase (TDO2), diverting tryptophan from serotonin synthesis toward the kynurenine pathway (13, 14). This metabolic shift alters glutamatergic neurotransmission via N-methyl-D-aspartate (NMDA) receptors, reduces the biosynthesis of monoaminergic neurotransmitters, and limits the availability of serotonin precursors, which may contribute to a decline in central serotonin levels and the onset of depressive symptoms (15). The production of pro-inflammatory cytokines is tightly regulated by a network of innate immune receptors and downstream signaling cascades (16). In both peripheral inflammatory diseases and depression, cytokine induction is primarily initiated through the activation of pattern recognition receptors (PRRs), including Toll-like receptors (TLRs) and NOD-like receptors (NLRs), which recognize pathogen-associated molecular patterns (PAMPs) and damage-associated molecular patterns (DAMPs) (17–19). Engagement of receptors such as TLR4 by lipopolysaccharide (LPS) or endogenous stress signals triggers intracellular signaling cascades involving NF-κB and MAPK pathways, leading to transcriptional activation of pro-inflammatory cytokines including IL-1β, IL-6, and TNF-α (20, 21). In parallel, activation of the NLRP3 inflammasome promotes caspase-1–mediated maturation and secretion of IL-1β and IL-18, further amplifying inflammatory responses (22, 23).

**Figure 1:**
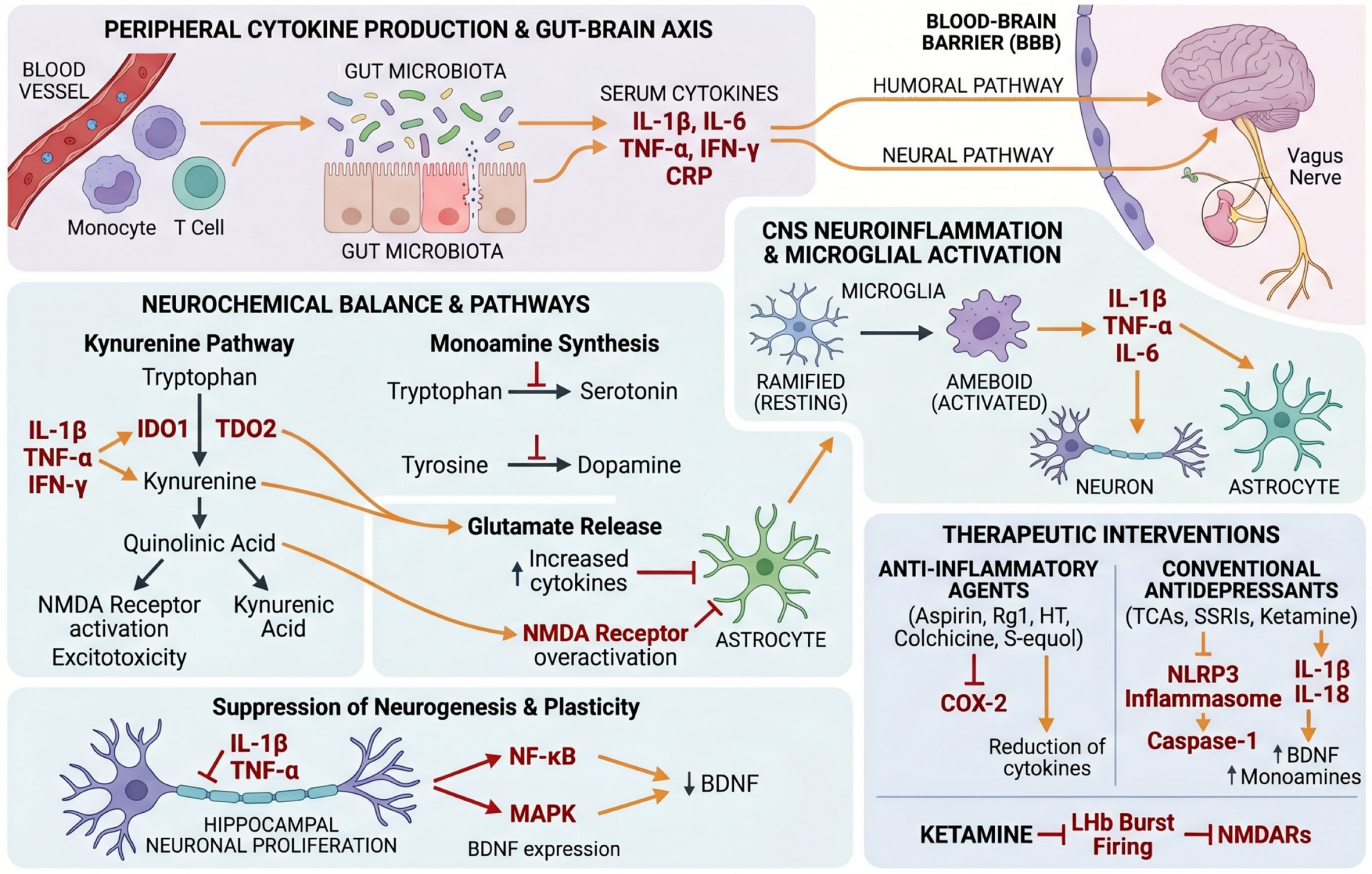
Serum inflammatory cytokines in the progression of depression.

At the cellular level, undifferentiated PC12 cells serve as a validated model for depression-related neuronal stress (24). Glucocorticoid exposure activates glucocorticoid receptors, reducing PC12 viability, impairing ATP production, and inducing DNA damage. These alterations coincide with upregulated IL-1β, IL-6, and TNF-α expression, which further compromise neuronal function and promote depressive phenotypes (25–27). The hippocampus, retaining adult neurogenic capacity via neural stem cell (NSC) proliferation, is particularly vulnerable to inflammatory disruption (28, 29). Hill et al. (30) demonstrated that activation of the G protein-coupled receptor GPR55 on murine NSCs can counteract IL-1β-induced suppression of neurogenesis *in vitro*. Given that hippocampal neurogenesis is essential for cognitive and behavioral regulation, reductions in neuronal proliferation may contribute to the manifestation of depressive symptoms. Microglia, the resident CNS immune cells, further shape the inflammatory milieu. Upon activation, microglia release pro-inflammatory cytokines that adversely affect neuronal viability and synaptic integrity, thereby propagating depressive pathology (31). These findings collectively suggest that both neurons and microglia mediate depression through the regulation of cytokine production and neuroimmune interactions (**Figure 1**).

## 3 Clinical correlates of inflammatory cytokines in depression

### 3.1 Inflammatory cytokines in depression

#### 3.1.1 Inflammatory cytokines and depression onset, course, and severity

Accumulating clinical evidence suggests that inflammatory cytokines are associated with the onset, progression, and severity of depression. In a cohort of 92 antidepressant-naïve depressed patients, Buspavanich et al. (32) reported that individuals with acute onset (≤4 weeks) exhibited significantly lower circulating levels of IL-2, IL-4, IL-6, IL-8, IL-10, TNF-α, and IFN-γ compared to those with insidious onset (>4 weeks). Similarly, patients with shorter illness duration (<6 months) showed reduced cytokine concentrations relative to those with prolonged episodes (6–24 months). Stratified analysis of 113 MDD patients further revealed a progressive elevation of inflammatory profiles with increasing depression severity, suggesting that the concentration of these inflammatory cytokines correlate with both the rapidity of onset and episode chronicity (33, 34). However, the inflammatory profile associated with depression appears to vary across demographic groups, particularly with age. For example, older adults (≥45 years) with depressive symptoms display greater interindividual variability in cytokine expression than non-depressed controls, without consistent elevation of mean levels. Notably, this variability is not clearly attributable to menopausal status, despite a higher proportion of female participants in symptomatic cohorts (35, 36). Moreover, several studies have linked depressive symptoms in middle-aged and older adults to an increased risk of cardiovascular disease, highlighting the broader systemic implications of inflammation in this population (37). These findings underscore the importance of early psychological screening and tailored interventions—particularly for females and aging individuals—to mitigate depression risk and support timely prevention strategies.

#### 3.1.2 Inflammatory cytokines and symptom dimensions, including suicidality

CRP, a downstream marker of systemic inflammation, has also emerged as a robust correlate of depressive symptomatology (38, 39). Serum CRP levels exceeding 3 mg/L are associated with heterogeneous manifestations—including low mood, appetite dysregulation, sleep disturbance, fatigue, and anxiety-related symptoms such as irritability and emotional lability (40, 41). Similarly, elevated IL-6 correlates with anhedonia, poor sleep quality, and appetite changes, while high circulating TNF-α predicts more severe presentations, pronounced pleasure loss, and reduced antidepressant responsiveness (42). Inflammatory cytokines may also serve as indicators of suicidality in depressed patients. Correlational analyses based on HAMD scores revealed that individuals with suicidal ideation exhibited significantly higher circulating levels compared to those without such ideation, implicating these cytokines in the neurobiology of suicide risk (43, 44). Postmortem analyses of hippocampal tissue from individuals who died by suicide further revealed pronounced elevations in IL-1, IL-6, IFN-γ, and TNF-α, which are hypothesized to intensify neuroinflammatory cascades through tryptophan–kynurenine pathway dysregulation (45, 46). Building upon these findings, Liu et al. (47) proposed that high levels of IL-1β and TNF-α may independently predict the risk of suicidal ideation and suicide attempts in depressed patients. Collectively, these studies underscore the critical involvement of inflammatory cytokines in modulating depression severity and suicidality.

### 3.2 Inflammatory cytokines and their neurocognitive and familial correlates

Emerging evidence positions inflammatory cytokines as mechanistic links between depression, neurocognitive dysfunction, and familial vulnerability. In an African American cohort, IL-6 significantly mediated the association between depressive symptom severity and impairments in psychomotor speed and cognitive flexibility (48). Individuals with more severe depressive symptoms exhibited slower visual scanning and reduced psychomotor performance, whereas those reporting frequent positive affect demonstrated better cognitive efficiency, lower serum IL-6 concentrations, and attenuated symptom burden (49, 50). These findings suggest that IL-6 may serve as a transdiagnostic biomarker bridging affective disturbance and executive dysfunction. Beyond individual-level associations, inflammatory profiles may reflect familial or shared environmental influences on mood disorder susceptibility. In a family-based study of 92 children and adolescents diagnosed with depression and/or anxiety undergoing fluoxetine treatment, Mevorach et al. (51) observed that non-pregnant mothers of affected youth exhibited significantly elevated plasma TNF-α levels compared to controls. Moreover, IL-1β concentrations in affected children positively correlated with maternal—but not paternal—cytokine levels, indicating potential maternal–child concordance in inflammatory regulation (52). This pattern suggests that hereditary factors, early-life environmental exposures, or maternal immune programming may jointly shape inflammatory trajectories relevant to mood disorder risk. Such insights may inform future efforts to develop personalized interventions that integrate inflammatory profiling with genetic and environmental risk assessments. However, the study design does not allow these influences to be clearly distinguished.

## 4 Serum inflammatory cytokines as therapeutic targets in depression

### 4.1 Anti-inflammatory bioactive compounds with antidepressant potential

Recent preclinical evidence highlights bioactive compounds with anti-inflammatory properties as promising therapeutic agents for depression. Naturally derived and synthetic agents—including ginsenoside Rg1, hydroxytyrosol (HT), benzothiophene N-hydroxyurea, colchicine, and S-equol—exert antidepressant-like effects in animal models by modulating neuroinflammation and suppressing pro-inflammatory cytokines (53, 54). Ginsenoside Rg1, a major bioactive component of *Panax ginseng*, exhibits low toxicity and notable neuroprotective properties (55, 56), significantly ameliorates depressive-like behaviors in chronic social defeat stress (CSDS) mice, as evidenced by improved performance in social interaction, sucrose preference (SPT), forced swim (FST), and tail suspension tests (TST) (55, 57). These behavioral improvements correlate with suppressed serum and hippocampal IL-1β, IL-6, and TNF-α levels and inhibited microglial activation, suggesting mediation via attenuated neuroinflammation (57).

Similarly, HT, a potent antioxidant and anti-inflammatory compound found in olive-derived polyphenols, alleviates depressive-like symptoms in chronic unpredictable mild stress (CUMS) models. HT treatment suppresses hippocampal microglial activation, reduces IL-1β and TNF-α concentrations, and upregulates BDNF, thereby restoring behavioral homeostasis (58). Benzothiophene N-hydroxyurea, a selective 5-lipoxygenase inhibitor, demonstrates efficacy in lipopolysaccharide (LPS)-induced models by reducing hippocampal neuroinflammation, lowering IL-1β and TNF-α levels, and attenuating neuronal apoptosis (59). Colchicine, a glycosylated quercetin derivative, decreases hippocampal IL-1β, IL-6, and TNF-α expression in CUMS-induced mice, reducing neuronal apoptosis and alleviating depressive behaviors through inhibition of pro-inflammatory signaling (60). S-equol, a soy isoflavone metabolite with established benefits for menopausal and metabolic conditions, reverses LPS-induced depressive-like behaviors in SPT, FST, and TST assays. It normalizes circulating IL-1β, IL-6, IL-10, and TNF-α levels and restores the hippocampal tryptophan–kynurenine balance, highlighting its capacity to mod both behavioral and biochemical markers of neuroinflammation (54). These studies underscore the therapeutic promise of anti-inflammatory compounds in modulating peripheral and central cytokine activity to exert antidepressant effects. Such agents may represent a novel pharmacological strategy for targeting inflammation-associated subtypes of depression.

### 4.2 Therapeutic approaches targeting serum inflammatory cytokines

#### 4.2.1 Conventional antidepressants with anti-inflammatory effects

Growing evidence indicates that several conventional antidepressants possess intrinsic anti-inflammatory properties, which may contribute to their therapeutic efficacy in depression. Among the classical antidepressants, tricyclic antidepressants (TCAs) and selective serotonin reuptake inhibitors (SSRIs) have been widely studied. Clomipramine and imipramine are representative TCAs, whereas citalopram and fluoxetine are identified as SSRIs (61, 62). *In vitro* studies have demonstrated that co-culture of immune cells with clomipramine or imipramine significantly reduced the release of IL-2 to approximately 60% of control levels, whereas citalopram exerted a weaker inhibitory effect (approximately 18%) (63). Following lipopolysaccharide (LPS) stimulation, all three agents markedly inhibit IL-1β and TNF-α production, with citalopram’s suppression of IL-6 approaching TCA efficacy under prolonged inflammatory challenge. These immunomodulatory actions align with clinical observations; meta-analyses confirm citalopram’s robust efficacy and favorable tolerability, suggesting that anti-inflammatory effects may partially underlie its therapeutic profile (64). Preclinical studies further support the anti-inflammatory and antidepressant effects of SSRIs. For instance, LPS administration in rodent models induces the release of IL-1β, IL-6, and TNF-α, leading to behavioral and neuroinflammatory changes that mimic depressive symptoms. These effects were ameliorated by SSRI treatment, underscoring their dual roles in modulating inflammation and mood (65).

Clinical mechanistic studies reinforce these findings. Alcocer-Gomez et al. (66) reported that untreated depressed patients exhibit elevated NLRP3 inflammasome expression, caspase-1 activation, and circulating IL-1β/IL-18 levels. Amitriptyline treatment significantly downregulated these inflammasome components and pro-inflammatory cytokines, indicating that NLRP3 pathway suppression constitutes a key pharmacodynamic mechanism. Similarly, fluoxetine reduces serum IL-1β and TNF-α concentrations in depressed patients, a finding corroborated by rodent studies demonstrating concurrent inhibition of JAK/STAT3 and TLR4/JNK signaling in macrophages (67, 68). These findings highlight serum inflammatory cytokines as viable targets for pharmacological intervention in depression. Antidepressants that modulate neuroinflammatory cascades may offer enhanced efficacy, particularly in inflammation-related subtypes of depression, and represent a promising direction for future drug development.

#### 4.2.2 Ketamine-based and adjunctive anti-inflammatory therapeutic strategies

In addition to traditional antidepressants, ketamine has emerged as a novel therapeutic agent with rapid and robust antidepressant effects, particularly in treatment-resistant depression (69). In a landmark study, Berman et al. (70) reported in 2000 the first clinical evidence of ketamine’s rapid antidepressant efficacy. Subsequent studies by Cui and colleagues (71, 72) confirmed ketamine’s potent, fast-acting, and sustained antidepressant properties. Mechanistically, ketamine inhibits burst firing in the lateral habenula (LHb) for up to 24 hours, thereby modulating downstream monoaminergic reward circuits. By blocking NMDARs, ketamine disrupts maladaptive excitatory neurotransmission and rapidly reverses depression-related behavioral and neurobiological abnormalities (73). Combination therapy with ketamine and docosahexaenoic acid (DHA), a polyunsaturated omega-3 fatty acid, has shown enhanced efficacy in murine models of depression. Co-treatment significantly reduced immobility time in behavioral despair tests (forced swim test), increased sucrose preference (an indicator of anhedonia recovery), and attenuated synaptic loss and morphological abnormalities in hippocampal neurons. Moreover, this combination markedly decreased levels of pro-inflammatory cytokines (IL-1β, IL-6, and TNF-α) in hippocampal and PC12 cells, while simultaneously increasing expression of BDNF. The treatment also inhibited LPS-induced serum inflammatory cytokines, suggesting that its antidepressant effects are mediated in part through suppression of neuroinflammation and enhancement of neurotrophic signaling (25). Collectively, current evidence demonstrates that a range of antidepressants—including clomipramine, imipramine, citalopram, amitriptyline, fluoxetine, and ketamine—not only exert antidepressant effects via classical neurotransmitter pathways but also modulate abnormal inflammatory cytokine profiles to varying degrees. These immunomodulatory actions highlight inflammation as a convergent therapeutic target in depression, providing a mechanistic rationale for anti-inflammatory interventions in future clinical strategies.

## Conclusion

Accumulating evidence indicates that serum inflammatory cytokines are not merely epiphenomena of depression, but integral components of its pathophysiological architecture. Cytokines such as IL-1β, IL-6, TNF-α, IFN-γ and CRP link peripheral immune activation to central neurobiological dysfunction through coordinated effects on innate immune signaling, tryptophan metabolism, monoaminergic transmission, microglial activation and hippocampal plasticity. Through pathways involving TLR4, NF-κB, MAPK, NLRP3 inflammasome activation, and IDO1/TDO2-dependent kynurenine metabolism, inflammatory signals can reshape neuronal homeostasis, weaken neurotrophic support and sustain maladaptive neuroimmune crosstalk. These mechanisms provide a coherent framework through which inflammation may influence symptom onset, illness chronicity, cognitive impairment and suicidality, thereby broadening current models of depression beyond monoamine-centered explanations.

Anti-inflammatory bioactive compounds, conventional antidepressants with immunomodulatory properties, and rapid-acting agents such as ketamine collectively suggest that inflammatory pathways are druggable and clinically relevant. However, substantial challenges remain, including marked interindividual heterogeneity, context-dependent cytokine signatures, and the difficulty of distinguishing causal inflammatory drivers from secondary disease-associated changes. Future progress will depend on longitudinal cohort studies, multi-omics integration, and stratified clinical trial designs that embed immune phenotyping into psychiatric research. A more precise delineation of cytokine-centered neuroimmune circuits may ultimately enable biomarker-guided diagnosis and more personalized therapeutic strategies for depression.
